# Cancer Research

**Published:** 1902-05-10

**Authors:** 


### The Hospital

A Journal of

The Medical Sciences and Hospital Administration.

Vol. XXXII.—No. 815. Edited by Sir Henry Burdett, K.C.B., and Solomon C. Smith, M.D., M.R.C.P. [May 10, 1902.

### Cancer Research.

The suggestion that a considerable sum of money should be contributed by the public, and committed to the hands of the Royal Colleges of Physicians and of Surgeons, to be by them employed in the conduct of a systematic research intended to elucidate the nature, causes, and cure of what is collectively called "cancer," appears already to have met with a sufficient degree of acceptance to be well on the road towards realisation. At the time of the last public statement on the question, something like £30,000 was already at the disposal of the colleges; and a letter has since appeared in the *Times*, from a correspondent signing himself "Research," who offers £5,000, on the condition that, within a specified time, a certain number of other donors of equal amount shall be forthcoming. Such a condition will almost certainly bring about its own fulfilment; and, indeed, the auspices under which the money is being asked for render it practically certain that enough will be contributed to justify the colleges in making a commencement of the great undertaking which they have in view. In such circumstances it becomes legitimate to inquire as to the methods of procedure which are likely to be adopted, and as to the degree of security to be afforded to donors on the important question of whether their contributions will be fruitfully or fruitlessly expended. The proposed undertaking is absolutely novel in its character, and hence there is no experience to which we can appeal with the object of throwing light upon the probabilities of its being carried to a successful issue. If nothing should come of it, or if the discoveries which we all desire should be the reward of independent and unsubsidised effort, while the subsidised inquirers were engaged in useless pottering, a very heavy blow would be struck at public confidence in the methods of scientific inquiry. If, on the other hand, the outcome of the research were even in a moderate degree successful, it would be universally felt that a new and powerful weapon had been forged as an aid in the perpetual medical battle against disease, and that no limits could be set to its future applicability and usefulness. We have no reason to doubt that these considerations will have their due weight with the respective governing bodies of the two colleges, but the stake at issue is of such vast importance that we should be neglecting our duty if we failed to call attention to them. Those governing bodies have not always been conspicuous for liberality or enlightenment; and their recent attitude towards the General Medical Council, on the question of the character and amount of training in physical science which is desirable as a part of medical education, has not been such as to add, in any material degree, to the confidence which we hope may be reposed in their fitness for the discharge of the entirely new duties which it is now proposed to commit to them.

The ordinary history of scientific discovery is that it has been the result of the patient industry of some single worker, who in most cases has been sustained against neglect, or even against ridicule and obloquy, by his own intense conviction of the trustworthiness of the gleams of light by which he was endeavouring to guide his steps. In opposition to the boundless credulity of complete ignorance, the credulity which has swallowed the assertions of "cancer-curers" and quacks, or which even now believes in the possession of an "infallible" remedy by some old woman in a remote village, it has not been uncommon to see the almost equally stupid incredulity of supposed knowledge, fortified against the intrusion of truth by the triple brass of self-conceit. Within a century of the present time a locomotive engine has been "scientifically" declared to be an impossibility, on the ground that its wheels would have no adhesion to the rails, and that they would spin round and round without communicating movement to the superstructure. When this objection was disposed of, it was said that movement through the air at the rate of five-and-twenty miles an hour would be fatal to the lives of passengers. A medical book of great reputation published only eight years ago contains a scoffing dismissal of the suggestion that Laveran's plasmodium could be connected with the disease of the fever patients in whose blood it had been discovered. The chief risk attending the proposed undertaking appears to us to depend upon the danger that a junta of worthy common-place men, fairly provided with the accepted knowledge of the day, and clear in their own minds as to the directions in which alone expansions of that knowledge may be looked for, should desire to exert a degree or kind of supervision which would be fatal to originality, and would provide for it only a cold shade instead of the fostering warmth under which it might be assisted to bring forth its fruits in due season. Against this danger the colleges are called upon most carefully to guard. Their business should be to assist capacity without obstructing it; and, while striving to avoid error in general, to be particularly upon their guard against errors which are "made in Germany."

